# Direct Mechanistic Evidence for a Nonheme Complex Reaction through a Multivariate XAS Analysis

**DOI:** 10.1021/acs.inorgchem.0c01132

**Published:** 2020-06-29

**Authors:** Francesco Tavani, Andrea Martini, Giorgio Capocasa, Stefano Di Stefano, Osvaldo Lanzalunga, Paola D’Angelo

**Affiliations:** † Dipartimento di Chimica, Università di Roma “La Sapienza”, P.le A. Moro 5, 00185 Roma, Italy; ‡ Dipartimento di Chimica, Università degli Studi di Torino, Via P. Giuria 7, 10125 Torino, Italy; § The Smart Materials Research Institute, Southern Federal University, Sladkova 178/24, 344090 Rostov-on-Don, Russia

## Abstract

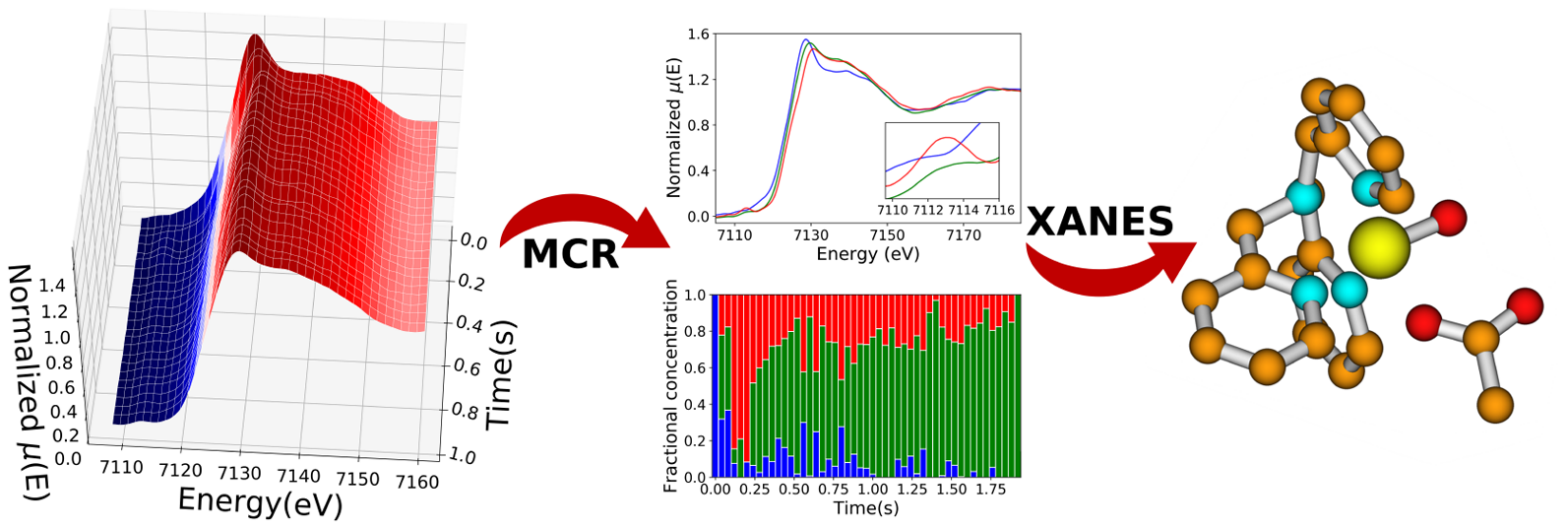

In this work, we propose a method to directly determine the mechanism of the reaction between the nonheme complex Fe^II^(tris(2-pyridylmethyl)amine) ([Fe^II^(TPA)(CH_3_CN)_2_]^2+^) and peracetic acid (AcOOH) in CH_3_CN, working at room temperature. A multivariate analysis is applied to the time-resolved coupled energy-dispersive X-ray absorption spectroscopy (EDXAS) reaction data, from which a set of spectral and concentration profiles for the reaction key species is derived. These “pure” extracted EDXAS spectra are then quantitatively characterized by full multiple scattering (MS) calculations. As a result, structural information for the elusive reaction intermediates [Fe^III^(TPA)(κ^2^-OOAc)]^2+^ and [Fe^IV^(TPA)(O)(X)]^+/2+^ is obtained, and it is suggested that X = AcO^–^ in opposition to X = CH_3_CN. The employed strategy is promising both for the spectroscopic characterization of reaction intermediates that are labile or silent to the conventional spectroscopic techniques, as well as for the mechanistic understanding of complex redox reactions involving organic substrates.

## Abstract 2

A combined multivariate and theoretical XAS analysis was proven to be a powerful method to obtain direct evidence for the mechanism of the reaction between the nonheme complex Fe^II^(tris(2-pyridymethyl)amine) ([Fe^II^(TPA)(CH_3_CN)_2_]^2+^) and peroxyacetic acid. This approach allowed to determine the time evolution of the concentration profiles for all reaction intermediates and to quantitatively characterize their structures, suggesting the sixth coordinating ligand of the nonheme oxo complex [Fe^IV^(TPA)(O)(X)]^+/2+^ is X = AcO^−^ in opposition to X = CH_3_CN.

## 1 Introduction

The full understanding of a given reaction mechanism, defined as the sequence of elementary steps leading reactants to products, is vital for chemical knowledge. In fact, unveiling the identity, the concentration time evolution, and the structural properties of the reaction intermediates provides essential insight into the process and paves the way for its rational optimization. Innovative experimental and theoretical approaches are required to tackle the complexity of chemical systems dealt with by contemporary researchers and to acquire accurate information on how these tranformations take place.

Nonheme iron complexes are a class of bioinspired catalysts that are gaining special interest for their capacity of oxidizing C–H and C=C bonds with high regio- and stereoselectivity.^1−3^ A special attention has been dedicated to the use of the environmentally friendly H_2_O_2_ oxidant in association with acetic acid, which is able to increase both catalytic activity and reaction selectivity. Under these conditions a metal-based oxidant is formed rather than free-diffusing radical species with the iron center that assumes different oxidation states during the reaction cycle.^4^ In a previous investigation, we employed time-resolved energy-dispersive X-ray absorption spectroscopy (EDXAS) to qualitatively identify the sequence of oxidation states during the reaction between the nonheme iron complex Fe^II^(tris(2-pyridylmethyl)amine) ([Fe^II^(TPA)(CH_3_CN)_2_]^2+^) and peroxyacetic acid (AcOOH) in CH_3_CN/AcOH (99.6:0.4 (v/v)) at 25 °C.^5^ Investigating this transformation at −40 °C, a seminal study showed that AcOOH oxidizes [Fe^II^(TPA)(CH_3_CN)_2_]^2+^ to the relatively stable oxo-complex [Fe^IV^(TPA)(O)(X)]^+/2+^, which in turn decays upon warming to the μ-oxo dimeric product [Fe_2_^III^(TPA)_2_(μ-O)(μ-OAc)]^3+^.^6^ The complex [Fe^IV^(TPA)(O)(X)]^+/2+^ was studied through a combination of electrospray ionization (ESI) mass spectrometry, UV–vis and Mössbauer spectroscopies, and an extended X-ray absorption fine structure (EXAFS) experiment, which however could not establish the identity of the sixth coordinating ligand X, maintaning X to be a molecule with a terminal oxygen or nitrogen atom bound to the Fe metal cation.^6^ In that same work, the authors advanced the hypothesis that the Fe(IV) species derived from an unobserved Fe(II)(TPA)-acyl peroxo complex. While the structures of the initial Fe(II) and final dimeric Fe(III) complex have been solved through X-ray crystallography some time ago,^7,8^ extensive spectroscopic studies have been performed to determine the true oxidation state and geometry of the reaction intermediate arising immediately after the initial Fe(II) species, but a definite answer has not yet been obtained. Talsi et al. measured new S = 1/2 electron paramagnetic resonance (EPR) signals at *g* = 2.71, 2.42, and 1.53 in the reaction of 40 mM [Fe^II^(TPA)(CH_3_CN)_2_]^2+^ in 1:1.7 CH_3_CN/CH_2_Cl_2_ with either H_2_O_2_/AcOH, peracetic acid or *m*-chloroperbenzoic acid at −60 °C.^9^ On the basis of this observation, the authors claimed to have identified a putative Fe^V^(O)(OAc) species, which has been predicted to be the true oxidant in the reaction of [Fe^II^(TPA)(CH_3_CN)_2_]^2+^ with C–H bond containing substrates.^4,10^ The same authors excluded the intermediate to be a low-spin acylperoxo complex of the kind [(L)Fe^III^(OOC(O)R)(S)]^2+^, because the EPR parameters of [(L)Fe^III^(OOR′)(S)]^2+^ coordination complexes are sensitive to the identity of the R′ group, and the same intermediate was observed in all reactions.^9^ However, this assignment was based on the isolation of the intermediate in only 7% yield, and it was not supported by additional spectroscopic characterizations.^4^ Subsequently, Que and co-workers replaced TPA with the variant TPA* (where six −CH_3_ and three −OCH_3_ groups substitute hydrogen atoms in the aromatic moieties of the three pyridine ligands of TPA) to generate a *g* = 2.7 intermediate in 50% yield at −40 °C.^4^ This strategy enabled the isolation and characterization of the intermediate as a low-spin acylperoxoiron(III) complex by combining UV–vis, EPR, resonance Raman, Mossbauer, and ESI-mass spectrometry data relative to the reactions of [Fe^II^(TPA)(CH_3_CN)_2_]^2+^ with either H_2_O_2_/AcOH, AcOOH, or *meta*-chloroperbenzoic acid (*m*CPBA). Specifically, the intermediate generated from the reaction of [Fe^II^(TPA*)(CH_3_CN)_2_]^2+^ with AcOOH yielded UV–vis and EPR spectra similar to those of the intermediate produced from the reactions of [Fe^II^(TPA*)(CH_3_CN)_2_]^2+^ with other oxidants, but it was not characterized by any other experimental techniques. The Fe(III) species was found to evolve to the corresponding Fe(IV)(TPA*)(O) complex in the reactions of [Fe^II^(TPA*)(CH_3_CN)_2_]^2+^ with H_2_O_2_/AcOH or AcOOH.

**Figure 1:**
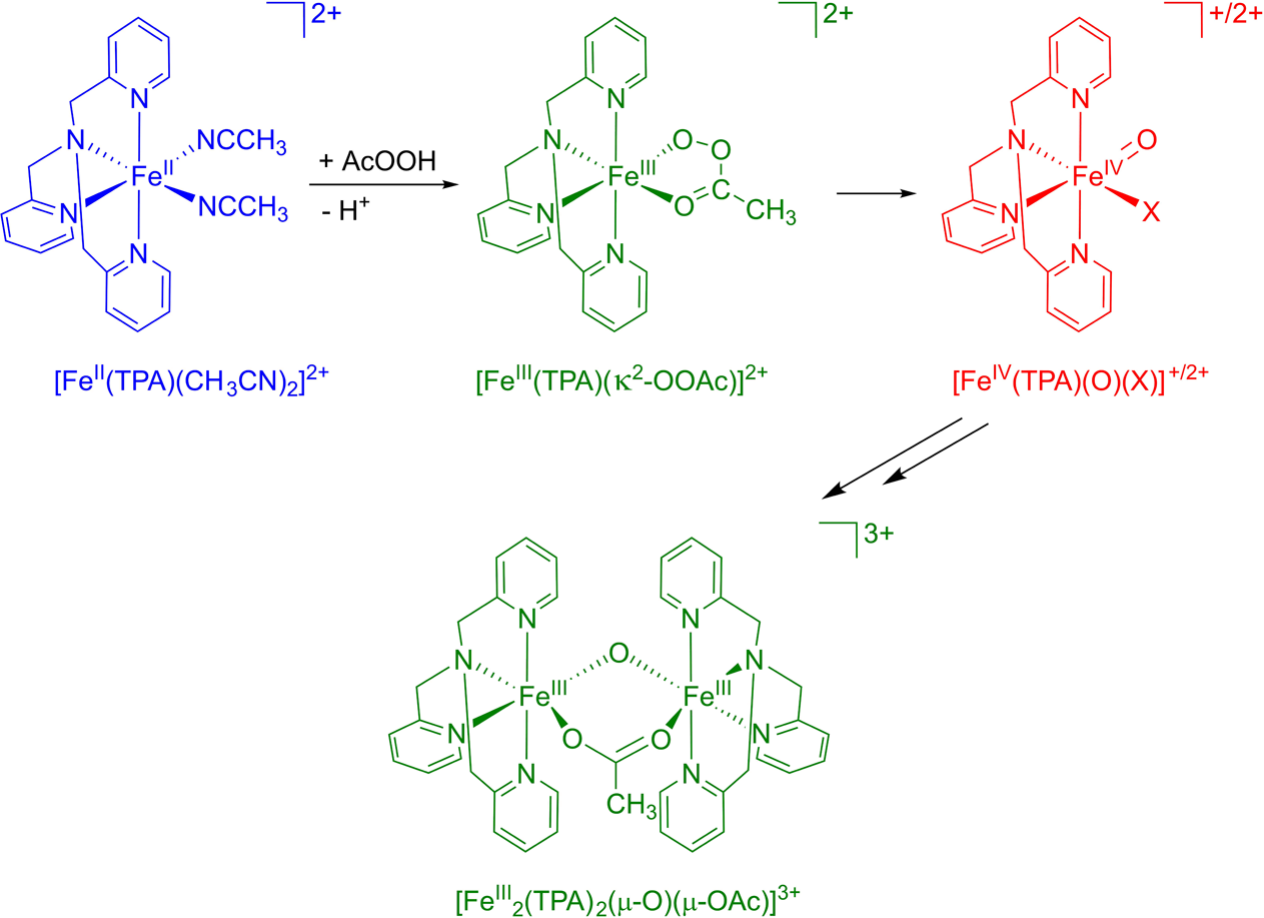
Proposed mechanism for the reaction between [Fe^II^(TPA)(CH_3_CN)_2_]^2+^ and AcOOH.

The proposed mechanism for the reaction between [Fe^II^(TPA)(CH_3_CN)_2_]^2+^ and AcOOH is presented in Figure 1.

Numerous spectroscopic techniques have been applied to follow fast chemical reactions with half–life times lower than seconds. Among them, X-ray absorption spectroscopy (XAS) is a unique and versatile tool^12^ that allows one to follow the variations in both the local electronic and structural configuration of a selected photoabsorbing atom.^11,13^ We recently used a coupled EDXAS/UV–vis approach to measure pseudo-first-order kinetic constants in a reaction involving a nonheme iron-oxo complex and a series of aromatic sulfides and benzyl alcohol, demonstrating the suitability of EDXAS to extract quantitative kinetic information for a bimolecular process on the millisecond to second time scale.^14^ Here, we show that it is possible to use a multivariate approach for the analysis of the EDXAS spectral data relative to the reaction of [Fe^II^(TPA)(CH_3_CN)_2_]^2+^ and AcOOH occurring at room temperature. This procedure enables one to extract the X-ray absorption near edge structure (XANES) spectra belonging to the reaction key species, to assess their oxidation states and lifetimes, and to quantitatively shed light on their elusive structures. The decomposition is achieved through a mathematical approach that belongs to the family of the Multivariate Curve Resolution (MCR) methods,^15−18^ a class of algorithms that has been applied extensively to the analysis of spectroscopic data coming from the monitoring of chemical reaction processes, such as UV–vis,^19−24^ fluorescence,^25−27^ nuclear magnetic resonance,^28−30^ circular dichroism,^31,32^ near-infrared (NIR),^33−36^ Fourier transform IR (FTIR),^37−39^ time-resolved FTIR,^40−42^ and Raman.^43,44^ MCR techniques have been increasingly applied also to time- and space-resolved XANES with studies investigating doped V_2_O_5_ lithium batteries,^45^ ZnO Q-dot formation,^46^ degradation of chlorine layered double hydroxide (LDH) upon heating,^47^ and a variety of catalytic systems in the solid phase.^48−53^ To the best of our knowledge, herein we report the first application of the MCR approach to XANES spectra pertaining to a bimolecular reaction in solution on organic substrates evolving on the millisecond time scale. In the presented framework, the direct in situ determination of the full mechanistic picture for the reaction involving the TPA substrate and the geometrical characterization of the reaction intermediates are achieved.

## 2 Materials and Methods

### 2.1 Materials

All reagents and solvents were employed at the highest commercial quality and used without additional purification. TPA and peracetic acid (36–40 wt % in acetic acid, stored at 4 °C) were purchased from Sigma-Aldrich. Iron(II) bis(trifluoromethanesulfonate)bis(acetonitrile), [Fe(OTf)_2_(CH_3_CN)_2_], was prepared according to a literature procedure from anhydrous Fe(II) chloride (Sigma-Aldrich).^54^ [Fe^II^(TPA)(CH_3_CN)_2_)](OTf)_2_ was prepared by metalation of the ligand TPA (Sigma-Aldrich) with [Fe(OTf)_2_(CH_3_CN)_2_] in dry CH_3_CN, and crystallization was performed by slow diffusion of dry diethyl ether in a dry dichloromethane solution as described in a literature method.^55^ Preparation and handling of air-sensitive materials were performed in an inert atmosphere by using a standard Schlenk and vacuum line techniques or a glove bag under N_2_ atmosphere. Subsequently, the complex was stored under inert atmosphere. When the [Fe^II^(TPA)(OTf)_2_] complex is dissolved in CH_3_CN, two solvent molecules enter the iron first coordination sphere giving rise to the [Fe^II^(TPA)(CH_3_CN)_2_)](OTf)_2_ complex. From now on, solely the cation [Fe^II^(TPA)(CH_3_CN)_2_]^2+^ will be mentioned.

### 2.2 Methods

#### 2.2.1 Reaction Details

For every stopped-flow mixing experiment, stock solutions of 70 mM [Fe^II^(TPA)(CH_3_CN)_2_]^2+^ in CH_3_CN and 70 mM AcOOH in CH_3_CN (diluted from the commercially available 36–40 wt % AcOOH solution in acetic acid, Sigma-Aldrich) were inserted into the reservoirs of the stopped-flow instrument. They were mixed in a 1:1 volume ratio at room temperature to obtain final concentrations of 35 mM for both [Fe^II^(TPA)(CH_3_CN)_2_]^2+^ and AcOOH. For all measurements, 100 μL of each solution was shot by the instrument into the cell.

#### 2.2.2 Energy Dispersive X-ray Absorption Measurements

EDXAS were collected at the ID24 beamline of the European Synchrotron Radiation Facility (ESRF), Grenoble (the ring energy was 6.0 GeV, and the current was 150–200 mA).^56^ The X-ray source consists of two undulators, whose gaps were tuned to place the first harmonic at 7100 eV. The beam was focused horizontally to an 8 μm full width at half-maximum (fwhm) spot on the sample by the curved Si(111) polychromator crystal in Bragg geometry. In the vertical direction, the beam was focused using a bent Si mirror at a glancing angle of 3 mrad with respect to the direct beam. To minimize sample radiation damage, the vertical spot size was set at 40 μm fwhm. Spectra were recorded in transmission mode using a fast read out low noise (FReLoN) high frame-rate detector based on charge coupled device (CCD) cameras optically coupled with a scintillator screen. Acquisition time was 40 ms for each spectrum. Sequences of 50–100 individual spectra were acquired, covering a total time span of 2–4 s during the reaction. Each sequence was repeated three times, and the data were averaged to obtain a better signal-to-noise ratio. The energy calibration was made by measuring the absorption spectrum of an Fe foil, and the first inflection point was set at 7111 eV. All measurements were performed at 25 °C. EDXAS spectra were recorded with a Bio-Logic SFM-400 stopped-flow device equipped with a flow-through quartz capillary cell. The quartz capillary cell had a diameter of 1.3 mm and wall thickness of ∼10 μm. The dead time of the stopped-flow device is ∼2.0 ms for the flow rate of 8 mL/s as calibrated using the procedure described elsewhere, and it defines the shortest kinetic time that is accessible for spectroscopic measurements.^5^

#### 2.2.3 EDXAS Data Treatment

The stopped-flow apparatus used to perform the reaction requires a quartz capillary cell that worsens the quality of the EDXAS spectra due to scattering by quartz. For each measurement the EDXAS spectrum of the cell containing pure acetonitrile was collected after the sample spectrum, using the same statistic. The cell spectrum was subtracted from the sample spectrum to gain a better signal-to-noise (S/N) ratio and a higher resolution for the structural oscillations and a more defined Fe K-edge position. The spectra were then subjected to a smoothing procedure using the Savitzky-Golay Smoothing filter, as described in refs (57 and 58).

#### 2.2.4 Decomposition of EDXAS Data into the Spectra and Relative Concentrations of Key Components

XANES time-resolved measurements yield a large series of spectroscopic data that may be arranged in a spectral matrix **D**, where each column of **D** is a spectrum measured at time *t*. Following the Lambert–Beer law, each experimental spectrum may be seen as the superposition of a number *N* of “pure” and uncorrelated components multiplied by their relative concentration.^17^ The decomposition of the experimental EDXAS data into the *N* spectra associated with the key reaction species and the relative concentration profiles was performed using the PyFitit code.^17^ To do so, this software employs a strategy belonging to the class of the MCR methods.

**Figure 2:**
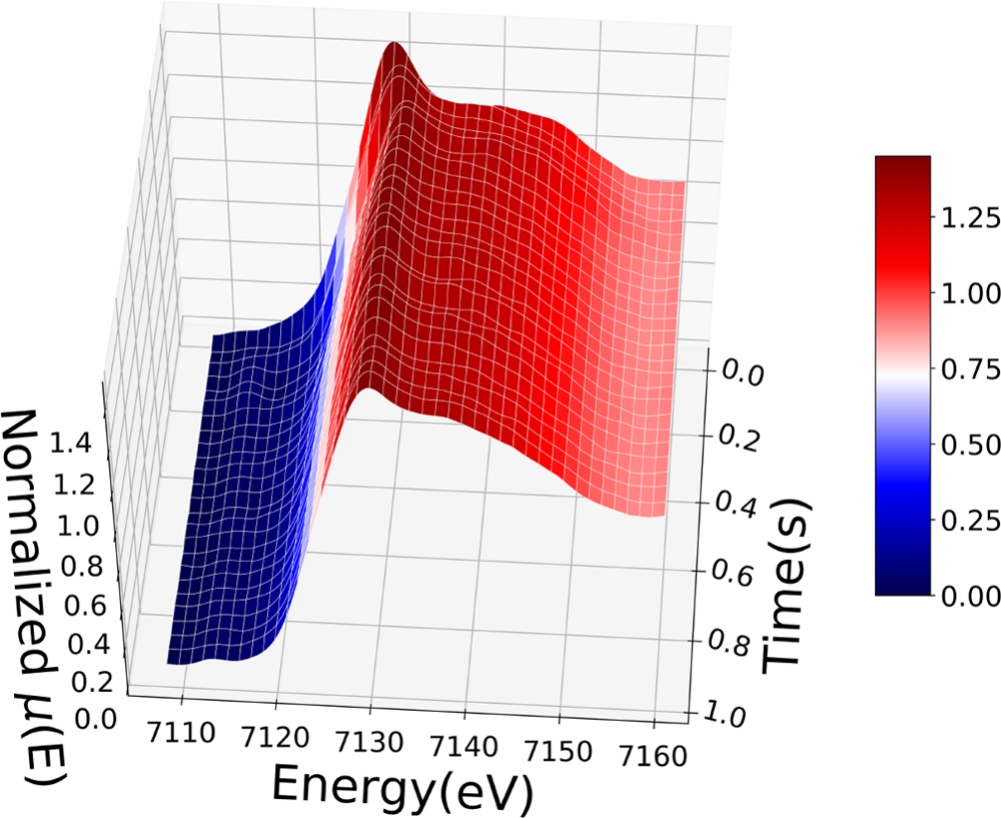
Time evolution of the Fe K-edge EDXAS spectra of complex [Fe^II^(TPA)(CH_3_CN)_2_]^2+^ (35 mM) recorded with a 40 ms time resolution after addition of AcOOH (35 mM) in CH_3_CN/AcOH (99.6:0.4 (v/v)) at room temperature. The first spectrum at *t* = 0.00 s is the EDXAS spectrum of a CH_3_CN solution containing the initial species [Fe^II^(TPA)(CH_3_CN)_2_]^2+^ (35 mM).

The decomposition’s starting point is the Singular Value Decomposition (SVD) expression:
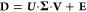
1where the product ***U*****Σ** contains, on its *N* columns, a set of values associable to the normalized absorption coefficients, **Σ** is a diagonal matrix called *singular values* term, whose elements are sorted in decreasing order, while **V** can be interpreted as the concentration matrix associated with the *N*-selected components. Finally, the error matrix **E** represents the lack of fit between the experimental data matrix **D** and the reconstructed one **μ** = ***U*****Σ*****V***. The SVD decomposition depends on the correct estimation of the number of components *N* present in the experimental spectral matrix. This may be achieved by combining different statistical and empirical evidence.^17^ Among them, in this work we chose to use the scree plot analysis as shown afterward in Figure 2a, since it is easily and effectively interpreted. At this stage, all matrices present in eq 1 are mere mathematical solutions of the spectral separation problem and do not possess any chemical meaning. Once *N* is established, the approach implemented by PyFitit requires the introduction of a transformation *N* × *N* matrix **T** in eq 1, using the relation **I** = **T T**^–1^
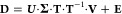
2where the spectra belonging to the key reaction species are given by **S** = ***U*****ΣT**, and their concentration profiles are given by **C** = **T**^–1^**V**. The matrix elements *T*_*ij*_ of matrix **T** are then modified by sliders to achieve **S** and **C**, which are chemically and physically interpretable. Once this step is achieved, one can finally write
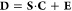
3

In this work, to reduce the unknown number of elements of **T**, which is in principle equal to *N*^2^, the normalization of all spectral components contained in matrix **S** and the mass balance condition for the concentrations contained in matrix **C** were imposed. Further, the first spectrum assigned to the reaction’s initial species, complex [Fe^II^(TPA)(CH_3_CN)_2_]^2+^, was constrained to be equal to the EDXAS spectrum recorded on a CH_3_CN solution containing complex [Fe^II^(TPA)(CH_3_CN)_2_]^2+^. This EDXAS spectrum is superimposable with the XAS spectrum recorded in transmission mode of a [Fe^II^(TPA)(CH_3_CN)_2_]^2+^ solution at 25 °C (see Figure 1 in ref (5)) and therefore belongs to the initial Fe(II) species. This spectral profile represents the *t* = 0 s starting point for the matricial decomposition. For the detailed explanation of how these constraints are imposed, see ref (17).

#### 2.2.5 XANES Data Analysis

Each XANES spectra extracted by the matrical decomposition was assigned to a reaction key species and analyzed using the MXAN code.^59,60^ This code is based on the calculation of theoretical spectra with a multiple scattering (MS) approach in the framework of the muffin tin (MT) approximation using a complex optical potential, exploiting the local density approximation of the excited photoelectron self-energy.^61−63^ The MT radii were calculated according to the Norman criterion. The self energy is calculated in the framework of the Hedin-Lundqvist (HL) scheme using only the real part of the HL potential, while an empirical approach is employed to account for inelastic losses in which the plasmon amplitude *A*_s_ and the energy onset *E*_s_ are refined.^64^ In all analyses the core hole lifetime Γ_c_ was kept fixed at 1.25 eV for Fe, while the experimental resolution Γ_res_ was optimized during the minimization procedure using a Gaussian function.

The analysis of the XANES spectra assigned to species [Fe^II^(TPA)(CH_3_CN)_2_]^2+^ was performed starting from an octahedral coordination model around the Fe atoms based on the crystallographic structure of the complex [Fe^II^(TPA)(CH_3_CN)_2_]^2+^.^7^ In this structure the Fe photoabsorber is coordinated by four nitrogen atoms belonging to the TPA backbone (N_TPA_) and by two CH_3_CN solvent nitrogen atoms N_ACN_. The minimization procedure of the Fe(II) species was performed by optimizing an Fe–N_TPA_ distance with a multiplicity of four and an Fe–N_ACN_ distance with a multiplicity of two. The geometry of the TPA ligand and of the acetonitrile molecules was kept fixed to the crystallographic initial structure.

The XANES calculations regarding complex [Fe^III^(TPA)(κ^2^-OOAc)]^2+^ were based on a previously reported density functional theory (DFT)-optimized molecular structure.^4^ In this complex, the central metal cation is coordinated to the four TPA nitrogen atoms and to a peracetate molecule through the negatively charged peracetate oxygen atom (O_per_) and the oxygen atom belonging to the acetate moiety (O_OAc_). The minimization procedure was applied by optimizing the Fe–N_TPA_ and the Fe–O_per_ distances, without altering the rest of the peracetate. The orientation of the peracetate was refined within a preset range of ±20° around the initial structure.

The MS analysis of [Fe^IV^(TPA)(O)(X)]^+/2+^ was performed using two different models. In the former (X = CH_3_CN) the minimization procedure was performed starting from the crystallographic structure of complex [Fe^II^(TPA)(CH_3_CN)_2_]^2+^, where one of the acetonitrile ligands was replaced by an oxygen atom (O_oxo_), and the Fe–N_ACN_ and Fe–O_oxo_ distances were optimized independently. In the latter (X = AcO^–^), the crystallographic structure around a single Fe atom of complex [Fe_2_^III^(TPA)_2_(μ-O)(μ-OAc)]^3+^ was employed as the initial geometry.^8^ In this case, the Fe–O_oxo_ and the Fe–O_OAc_ distances were independently optimized. For complexes [Fe^IV^(TPA)(O)(CH_3_CN)]^2+^ and [Fe^IV^(TPA)(O)(OAc)]^+^ the orientations of the oxo atom, of the CH_3_CN molecule, and of the acetate group were varied within a preset range of ±20° around the initial geometries.

The analysis of the XANES spectrum assigned to complex [Fe_2_^III^(TPA)_2_(μ-O)(μ-OAc)]^3+^ was performed starting from its crystal structure.^8^ In this structure there are two Fe atoms each coordinated by a TPA ligand and an oxygen atom belonging to an acetate molecule, and they are linked through a bridging oxygen atom (O_bridge_). Because of the symmetry of the two Fe sites, the minimization procedure was performed by optimizing three bond lenghts (Fe–N_TPA_, Fe–O_OAc_, and Fe–O_bridge_). Theoretical XANES spectra were calculated including scatterers within 5 and 6 Å around a selected Fe atom, and it was found that scattering atoms do not contribute significantly to the theoretical spectrum outside a cutoff radius of 5 Å.

Hydrogen atoms were not included in all MXAN analyses. For all spectra, five nonstructural parameters were refined, namely, the threshold energy *E*_0_, the Fermi energy level *E*_F_, the energy and amplitude of the plasmon *E*_s_ and *A*_s_, and the experimental resolution Γ_res_. The quality of the fits was estimated with the residual function *R*_sq_.^59−61^

## 3 Results and Discussion

Figure 2 shows the experimental EDXAS spectra recorded with a time resolution of 40 ms during the reaction of [Fe^II^(TPA)(CH_3_CN)_2_]^2+^ (35 mM) with AcOOH (35 mM) CH_3_CN/AcOH (99.6:0.4 (v/v)) at 25 °C, where the *t* = 0.00 s spectrum was fixed to the EDXAS spectrum of a CH_3_CN solution containing [Fe^II^(TPA)(CH_3_CN)_2_]^2+^ (35 mM). One can note that the most apparent variations in the spectra are contained in the spectra between *t* = 0.00 s and *t* = 0.40 s from reaction start. Notably, between *t* = 0.04 s and *t* = 0.20 s the energy edge progressively shifts to higher energies, while between *t* = 0.20 s and *t* = 0.40 s the energy edge moves to lower energies. In this same time interval one may note the appearance of a 1s → 3d transition located at ∼7113 eV. This transition is visible for spectra between *t* = 0.12 s and *t* = 0.20 s before decaying to zero as the reaction proceeds. After *t* = 0.60 s, the visible spectral variations are greatly abated. These results are consistent with the reaction mechanism shown in Figure 1, where the initial Fe(II) species undergoes a first oxidation to the Fe(III) complex, which is further oxidized to the Fe(IV) oxo complex, which returns by decay to an Fe(III) state. Iron acquires three different oxidation states (assigned to complexes stable enough to be isolated) during the reaction, and therefore one expects the number *N* of independent components present in the data mixture to be *N* = 3 or greater.

**Figure 3:**
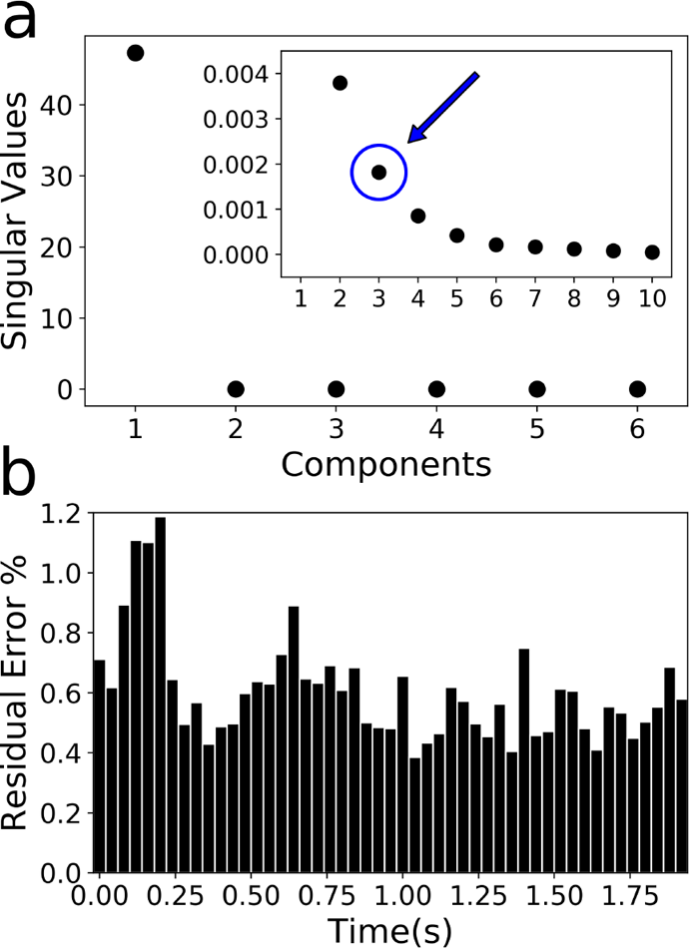
Results obtained from the PCA analysis. (a) Scree plot: plot of the singular values derived from the SVD decomposition against the related component number. (inset) A magnification where the singular value relative to PC = 3 is highlighted by a blue circle and arrow. (b) Residual percentage error associated with the reconstruction of the experimental EDXAS data set with *N* = 3 components.

Principal component analysis (PCA) was applied to the EDXAS data set to confirm this qualitative analysis and to identify the number of chemical components present in the reaction data mixture.^65^ The results are presented in Figure 3. The singular values, extracted from SVD method, are the diagonal elements of matrix **Σ** reported in eq 1. These quantities are proportional to the data variance explained by each component. It follows that each of them can be properly plotted against the related component number, generating the so-called scree plot, as reported in Figure 3a. One can note from the plot the existence of an elbow indicating the presence of three relevant components. Conversely, for numbers of components greater than three, the related singular values decrease slowly with approximately the same decaying slope, indicating that these components contribute to the data set reconstruction in the same way and are, for this reason, associated with noise. This statistical evidence suggests there are three principal components present in the data set. This result is in accordance with the chemical knowledge of the reaction mechanism that predicts the succession of three distinct oxidations states for Fe. The percentage residual error committed in reconstructing the data set with three components is shown in Figure 3b. The percentage error function was calculated with the following expression
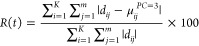
4where *d*_*ij*_ and μ_*ij*_^PC=3^ are the normalized absorbance values for the data set and for the data set reconstructed with *N* = 3, respectively (*K* and *m* represent the number of acquired time-resolved spectra and of the energy points, respectively). Interestingly, one may observe an increase in the percentage error in proximity of the spectra recorded between *t* = 0.04 s and *t* = 0.16 s. Since the main EDXAS spectral variation in the experimental data is observed in the same time interval, this finding suggests the presence of a diluted and transient species that contributes in small percentage to the overall measured signal. It is probable that by including an ulterior fourth (or fifth) component in the decomposition this error would diminish. However, relying on the knowledge of the reaction mechanism, on the scree plot analysis, and on the relatively small error (inferior to 1.2%) committed in the reconstruction with *N* = 3, we decided to employ only three PCs for the subsequent analysis.

The transformation matrix approach implemented in PyFitit^17^ was used to decompose the data set, employing a 3 × 3 **T** transformation matrix. Furthermore, by imposing the set of constraints described in Section 2.2.4, the number of *T*_*ij*_ elements was reduced from nine to four. Each of these four terms was varied preserving the mass balance condition and the non-negativity of the extracted spectra and concentration profiles. A solution to the decomposition expressed by eq 3, possessing a sound chemical meaning, was achieved through the matrix
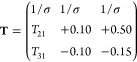
5where σ is the normalization coefficient, 1/σ = −0.17, *T*_21_ = 0.51, and *T*_31_ = 0.57.

**Figure 4:**
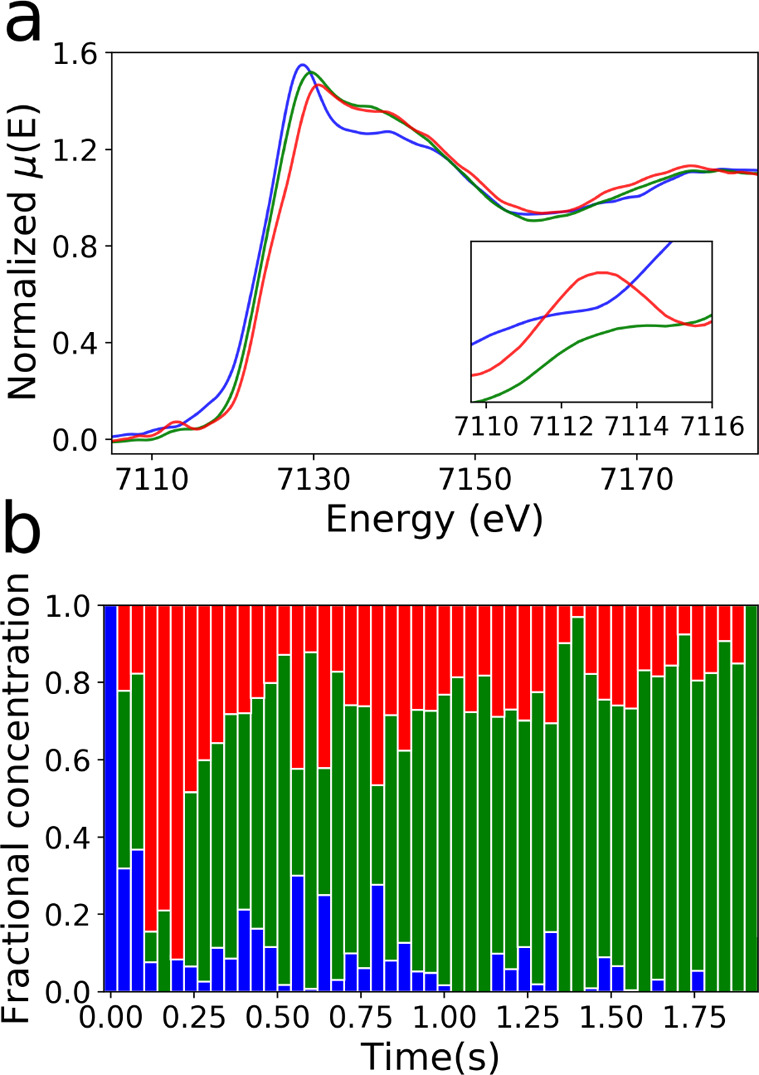
Fe K-edge XANES spectra (a) and fractional concentration profiles (b) extracted by using the transformation matrix-based decomposition.

Figure 4a shows the isolated EDXAS spectra, and Figure 4b shows their fractional components in the reaction mixture. The first spectral component (blue) belongs to complex [Fe^II^(TPA)(CH_3_CN)_2_]^2+^. The second (red) and third (green) components are assigned to complexes in which iron has the oxidation states of Fe(IV) and Fe(III), respectively. In fact, the oxidation state of each spectrum is identified by the relative energy position of the main absorption edge. The first inflection point of the spectrum belonging to the initial Fe(II) reactant lies at lower energy than those assigned to the Fe(IV) and Fe(III) species, while that of the Fe(IV) is found at the highest energies. Interestingly, the Fe(IV) complex shows a 1s → 3d dipole-forbidden transition centered at ∼7113 eV. This feature is absent in the spectrum of the Fe(II) reactant and weak in that of the Fe(III) compound. This finding further supports the proposed identification of the reaction species. It is known that Fe(IV) oxo complexes show a relatively intense 1s → 3d transition due to their noncentrosymmetry, and it has been reported that this is also the case for complex [Fe^IV^(TPA)(O)(X)]^+/2+^.^6^ On this basis, the second XANES spectral component is assigned to complex [Fe^IV^(TPA)(O)(X)]^+/2+^.

Looking at Figure 4b one may note that the fractional concentration of the initial [Fe^II^(TPA)(CH_3_CN)_2_]^2+^ complex rapidly decays to zero, while the concentration of the Fe(IV) species shows an accumulation between *t* = 0.12 s and *t* = 0.20 s. Conversely, the concentration of the Fe(III) component is prevalent before the formation of the oxo complex at *t* = 0.04 s and *t* = 0.08 s. It decreases to almost zero when the concentration of Fe(IV) reaches its maximum, and then it gradually increases to become the reaction product from *t* = 0.24 s until the end of the process. These results confirm the sequence of the oxidation states that Fe assumes during the reaction shown in Figure 1 and prove, through the direct analysis of room-temperature reaction EDXAS spectra, that the starting Fe(II) species initially evolves to an Fe(III) intermediate.

The transformation matrix-based approach implemented in this investigation inherently suffers of rotational ambiguity. It follows that the solutions of the decomposition problem shown in Figure 4 are not unique.^15,17^ To address the validity of the extracted spectra and concentrations for the reaction components, the time evolution of the area belonging to the pre-edge 1s → 3d transition at 7113 eV and of the edge energy position of the EDXAS spectra were evaluated turning to the raw EDXAS time-resolved spectra.

**Figure 5:**
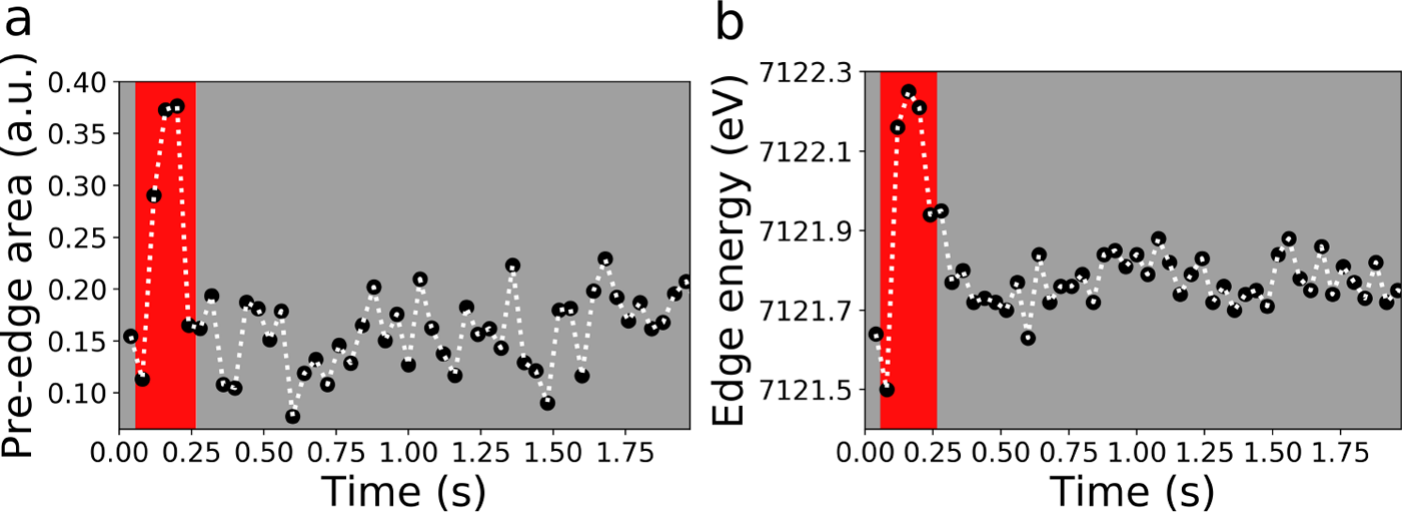
Time evolution of the area (in arbitrary units) of the 1s → 3d transition located at 7113 eV (a) and of the edge energy evaluated at a height of μ(*E*) = 0.40 (b).

Figure 5a presents the variation during the reaction of the area of the dipole-forbidden transition measured on the raw XANES spectra. One can note that there is a maximum localized at *t* = 0.16 s, which is indicative of the formation of the noncentrosymmetric oxo complex. The time evolution of the Fe K-edge energy (shown Figure 5b) was qualitatively evaluated by measuring the energy at μ(*E*) = 0.40 for each raw spectrum, an approach that we have shown to be successful in the analysis of EDXAS spectra acquired during a chemical reaction in solution.^14^ The result of this procedure is shown in Figure 5b, where one can note a shift of the main absorption edge to higher energies of ∼0.8 eV from *t* = 0.08 s to *t* = 0.12 s. Such a change is consistent with the oxidation of the Fe(III) present in the reaction mixture to Fe(IV). This analysis confirms also that the lifetime of the Fe(IV) complex is in the range within *t* = 0.12 s and *t* = 0.24 s. Finally, the edge moves to lower energies upon the reduction of Fe(IV) to Fe(III) and reaches a constant value, which does not exactly coincide with the edge value prior to the Fe(III) → Fe(IV) oxidation. This is due to the presence at *t* = 0.04 s and *t* = 0.08 s of the Fe(II) component and to the contribution of the Fe(IV) component at *t* > 0.24 s. Such independent methods fully support the validity of the mathematical solutions shown in Figure 4.

On the basis of the established reaction mechanism, the isolated spectral component associated with an Fe(III) oxidation state may be assigned to both the proposed acyl-peroxo intermediate [Fe^III^(TPA)(κ^2^-OOAc)]^2+^ and to the dimeric product [Fe_2_^III^(TPA)_2_(μ-O)(μ-OAc)]^3+^. In fact, the local geometry around the central Fe(III), in both complexes, is made of four nitrogen atoms belonging to the TPA chain and of two coordinating oxygen atoms. Further, as is listed in Table 1 the DFT-optimized first-shell distances of complex [Fe^III^(TPA)(κ^2^-OOAc)]^2+^ and the crystallographic first-shell distances of complex [Fe_2_^III^(TPA)_2_(μ-O)(μ-OAc)]^3+^ closely resemble one another. This evidence, together with the relatively small error committed in reproducing the experimental data set with *N* = 3, supports the identification of the same spectral component for both Fe(III) species.

**Table 1:** Fe K-Edge XANES Best-Fit Structural Parameters^a^

	Fe–N_TPA_		Fe–N_ACN_		Fe–O_oxo_		Fe–O_per_		Fe–O_OAc_		Fe–O_bridge_	
	*N*	*R* (Å)	*N*	*R* (Å)	*N*	*R* (Å)	*N*	*R* (Å)	*N*	*R* (Å)	*N*	*R* (Å)
**1**												
cryst^7^	4	1.91(1) – 1.99(1)	2	1.92(1) – 1.93(1)								
this work	4	2.04(5)	2	1.97(5)								
**2**												
DFT^4^	4	1.94–2.01					1	2.00	1	1.81		
this work	4	2.03(5)					1	2.00(5)	1	1.86(5)		
**3**												
EXAFS^6^	4	1.99	1^b^	2.20^b^	1	1.67(2)			1^b^	2.20^b^		
**3a**												
this work	4	2.04(5)			1	1.77(5)			1	2.05(5)		
**3b**												
this work	4	2.05(5)	1	2.06(5)	1	1.77(5)						
**4**												
cryst^8^	8	2.105(6) – 2.242(6)							2	1.972(6) – 2.038(6)	2	1.779(3)–1.815(3)
this work	8	2.06(5)							2	1.91(5)	2	1.84(5)

a Fe K-edge XANES best-fit structural parameters of [Fe^II^(TPA)(CH_3_CN)_2_]^2+^ (**1**), [Fe^III^(TPA)(κ^2^-OOAc)]^2+^ (**2**), [Fe^IV^(TPA)(O)(X)]^+/2+^ (**3**), [Fe^IV^(TPA)(O)(OAc)]^+^ (**3a**), [Fe^IV^(TPA)(O)(CH_3_CN)]^2+^ (**3b**), and [Fe_2_^III^(TPA)_2_(μ-O)(μ-OAc)]^3+^ (**4**) compared to the available literature: crystallographic, EXAFS, and DFT data.  and  are the average distances between the metal cation, the TPA, and the solvent nitrogen atoms, respectively,  is the distance between the metal cation and the coordinating oxygen of the oxo group in Fe^IV^(TPA)(O)(OAc)]^+^ and [Fe^IV^(TPA)(O)(CH_3_CN)]^2+^,  is the distance between Fe and the negatively charged peracetate oxygen in complex [Fe^III^(TPA)(*k*^2^-OOAc)]^2+^,  is the average distance between the metal cation and the acetate group, and  is the average distance between the metal cation and the bridging oxygen atom in complex [Fe_2_^III^(TPA)_2_(μ-O)(μ-OAc)]^3+^, while *N* is the coordination number.

b A nitrogen or an oxygen atom located at 2.20 Å from the central cation is reported in ref (6).

**Figure 6:**
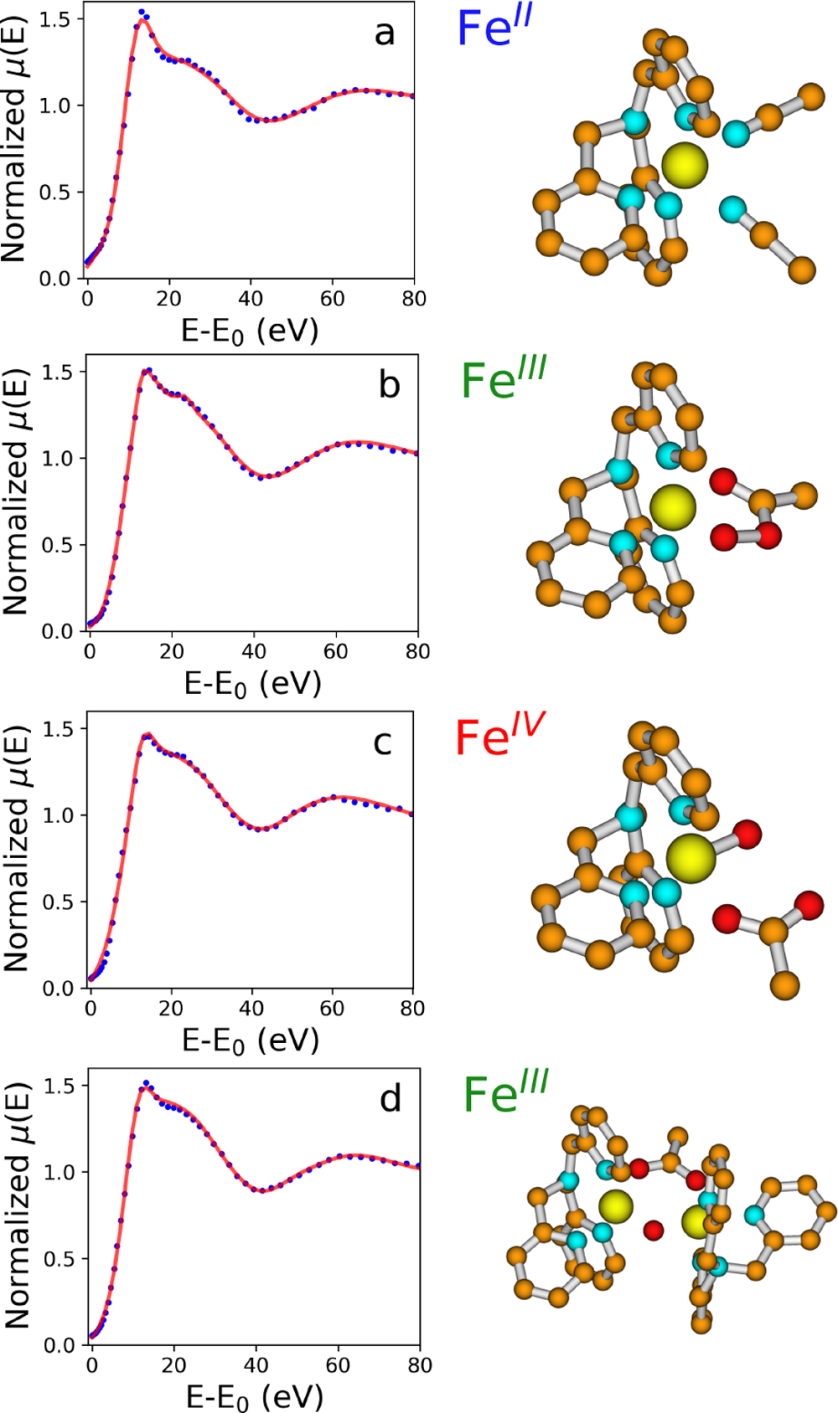
Fe K-edge XANES spectra (blue, dotted lines) of complex [Fe^II^(TPA)(CH_3_CN)_2_]^2+^ (a) and assigned to complexes [Fe^III^(TPA)(κ^2^-OOAc)]^2+^ (b), [Fe^IV^(TPA)(O)(OAc)]^+^ (c), and [Fe_2_^III^(TPA)_2_(μ-O)(μ-OAc)]^3+^ (d) compared with the theoretical curves (red, full lines) calculated with optimized geometrical models. The associated molecular clusters are also depicted, where iron, nitrogen, carbon, and oxygen atoms are in yellow, cyan, orange, and red, respectively.

To test these hypotheses and to obtain quantitative structural information regarding all the reaction intermediates, a full MS analysis was performed on the three isolated spectral components. The XANES spectrum of the Fe(II) complex is quite different from those of the Fe(III) and Fe(IV) species. As previously mentioned, the Fe first coordination shell is made up by the four nitrogen belonging to the TPA backbone, which were placed at the same Fe–N_TPA_ distance, and by two nitrogen atoms belonging to the CH_3_CN solvent molecules. During the fitting procedure, the Fe–N_TPA_ and the Fe–N_ACN_ distances were refined together with the nonstructural parameters to obtain the best agreement with the experimental spectrum. The best-fit results are shown in Figure 6a, while the molecular cluster obtained from the minimization is shown to the right. The agreement between the theoretical spectrum and the isolated component is excellent. The refined parameters are listed in Table 1. The Fe–N_TPA_ and the Fe–N_ACN_ distances are in good agreement with the crystallographic values within the statistical errors. It is well-known that systematic errors are present in the XANES analysis performed with MXAN and that they arise mostly because of the poor approximation used for the phenomenological broadening function Γ(*E*) that mimics the electronic damping. In all cases studied until now such systematic errors did not appreciably affect the structural results, confirming how this spectroscopy is dominated by the geometry of the atomic cluster rather than by its electronic structure.^66−69^ The full list of nonstructural parameters is reported in Table 2.

**Table 2:** Nonstructural Parameters^a^

	*E*_0_ (eV)	*E*_F_ (eV)	Γ_exp_	*E*_s_ (eV)	*A*_s_	*R*_sq_
**1**	–4.8	–0.8	2.0	15.6	11.8	2.0
**2**	–4.3	2.9	1.0	24.5	11.8	1.1
**3a**	–3.2	–0.2	1.3	15.5	8.1	2.8
**3b**	–4.0	–2.1	1.9	25.0	10.8	2.9
**4**	–3.5	2.8	1.9	18.7	7.4	1.3

a Nonstructural parameters obtained from the MXAN analysis of the Fe K-edge XANES spectra of [Fe^II^(TPA)(CH_3_CN)_2_]^2+^ (**1**), [Fe^III^(TPA)(κ^2^-OOAc)]^2+^ (**2**), [Fe^IV^(TPA)(O)(OAc)]^+^ (**3a**), [Fe^IV^(TPA)(O)(CH_3_CN)]^2+^ (**3b**), and [Fe_2_^III^(TPA)_2_(μ-O)(μ-OAc)]^3+^ (**4**). *E*_0_ is the threshold energy, *E*_F_ is the Fermi energy level, *E*_s_ and *A*_s_ are the energy and amplitude of the plasmon, Γ_res_ is the experimental resolution, and *R*_sq_ is the residual function.

As far as the Fe(IV) oxo complex is concerned, the MXAN analysis was performed using two different models. In the former ([Fe^IV^(TPA)(O)(OAc)]^+^) the central Fe cation is coordinated to an acetate molecule and to the TPA chain. In this case, the Fe–O_oxo_ and the Fe–O_OAc_ distances were optimized independently together with the Fe–N_TPA_ one. The results of this analysis are shown in Figure 6c, while the best-fit structural parameters are listed in Table 1. Also in this case the agreement between the experimental and theoretical spectra is satisfactory (*R*_sq_ = 2.8), suggesting that an acetate molecule coordinates the Fe atom in the Fe(IV) species.

**Figure 7:**
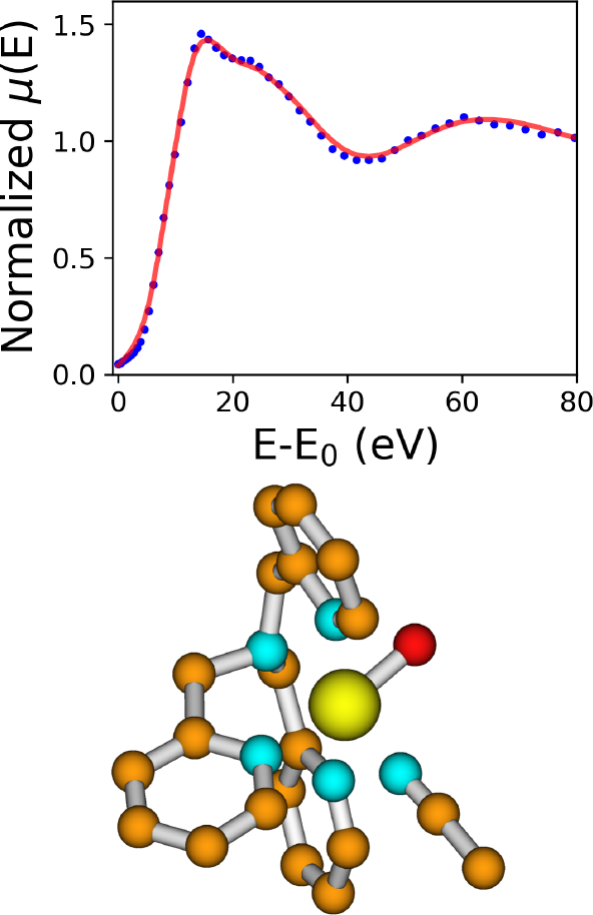
Fe K-edge XANES isolated spectrum relative to the [Fe^IV^(TPA)(O)(X)]^+/2+^ complex (blue, dotted line) compared with the theoretical curve (red, full line) calculated upon an optimized geometrical model, where X = CH_3_CN. The best-fit geometry is also depicted, where iron, nitrogen, carbon, and oxygen atoms are in yellow, cyan, orange, and red, respectively.

Further proof of this hypothesis was gained by performing a second minimization using a structural model where the central Fe is coordinated to the four TPA nitrogens, the oxo oxygen atom, and a CH_3_CN ligand. In this case the Fe–O_oxo_ and the Fe–N_ACN_ distances were optimized together with the Fe–N_TPA_ bond length. The best-fit structural and nonstructural parameters are listed in Tables 1 and 2, respectively. The comparison of the theoretical and isolated XANES spectra is presented in Figure 7, while the corresponding optimized atomic cluster is depicted below. In this case a slightly worse agreement was obtained between the two XANES spectra (*R*_sq_ = 2.9). This finding supports the hypothesis that a molecule coordinating the central Fe cation with an oxygen atom, such as acetate, has a higher residence time compared to that of a molecule coordinating the metal site with a nitrogen atom, such as CH_3_CN. Consequently, one may suggest that the previously unidentified sixth ligand in the [Fe^IV^(TPA)(O)(X)]^+/2+^ complex is the acetate anion.

Through the quantitative analysis of the XANES spectrum extracted from the decomposition, identical first-shell distances are found for both X = AcO^–^ and X = CH_3_CN, as reported in Table 1. The Fe–N_TPA_ distance is 2.04 Å, a value that coincides within the experimental errors with the EXAFS reported distance for complex [Fe^IV^(TPA)(O)(X)]^+/2+^.^6^ Additionally, the theoretical calculations derive a distance between the central Fe cation and the sixth coordinating ligand of 2.05(5) and 2.06(5) Å for X = AcO^–^ and X = CH_3_CN, respectively, to be compared with the literature value of 2.20 Å. Notably, the minimization calculations yielded an Fe–O_oxo_ distance of 1.77(5) Å. This value is at least 0.03 Å higher than the previously reported Fe–O_oxo_ distance for complex [Fe^IV^(TPA)(O)(X)]^+/2+^, which amounts to 1.67(2) Å.^6^ Additionally, the Fe–O_oxo_ distance was evaluated through X-ray crystallography for some nonheme oxo complexes such as [Fe^IV^(TMC)(O)(NCCH_3_)]^2+^ and [Fe^IV^(N4Py)(O)]^2+^, and it was found to be 1.646(3)^70^ and 1.639(5) Å,^71^ respectively. Numerous other nonheme oxo complexes have been characterized through EXAFS, with the Fe–O distance residing in the range between 1.62 and 1.70 Å.^72^ This slight discrepancy of our results with the existing literature may be because the 1.77(5) Å value is obtained on the direct analysis of the spectrum at room temperature of the Fe(IV) species, whereas other measurements have been all performed at low temperatures on a frozen solution or on the crystal, if available. Moreover, as previously underlined, a systematic error is present in the structural determinations conducted with MXAN, as evidenced in the geometrical characterization of other iron heme complexes and heme proteins.^66^

The XANES spectrum assigned to complexes [Fe^III^(TPA)(κ^2^-OOAc)]^2+^ and [Fe_2_^III^(TPA)_2_(μ-O)(μ-OAc)]^3+^ was subjected to two distinct minimization procedures. In the first one, the spectrum was analyzed starting from the DFT-optimized structure^4^ associated with complex [Fe^III^(TPA)(κ^2^-OOAc)]^2+^, where the Fe cation is coordinated by the four nitrogens of the TPA chain and by two oxygen atoms belonging to a peracetate molecule. The results of the analysis are shown in Figure 6b, where the experimental and theoretical curves are reported together with the molecular cluster. The agreement between the data is excellent (*R*_sq_ = 1.1) and the structural results coincide with the literature data within the statistical errors (Table 1). These findings represent important structural data that confirm the identity of the reaction intermediate [Fe^III^(TPA)(κ^2^-OOAc)]^2+^. Note that a small percentage of the corresponding Fe(V) oxo complex may form upon heterolysis of the O–O bond of the Fe(III) peroxo species as observed at low temperature by Talsi et al.^9,73^ However, at room temperature the Fe(V) species is too unstable to be observed by our method given the time scale of our experimental conditions.^4,74^

Finally, the XANES spectrum was analyzed starting from the crystal structure of the μ-oxo dimeric species [Fe_2_^III^(TPA)_2_(μ-O)(μ-OAc)]^3+^.^8^ The same coordination environment was used for both Fe^III^ atoms in the dimer. It comprises the four nitrogen atoms of the TPA ligand, the acetate molecule, and a bridging oxygen atom. During the fitting procedure, the Fe–N_TPA_, Fe–O_OAc_, and Fe–O_bridge_ distances were optimized, while the TPA structure was kept fixed to the initial geometry, and all of the atoms within 5 Å of the central metal cation were included in the theoretical calculation. Figure 6d presents the experimental and theoretical spectra together with the atomic cluster. The agreement between the two curves is very good (*R*_sq_ = 1.3). The structural results, listed in Table 1, highlight a slight compression of the Fe–N_TPA_ and Fe–O_OAc_ bond lengths compared to the crystal structure. One may note that the calculated first-shell distances for complexes [Fe^III^(TPA)(κ^2^-OOAc)]^2+^ and [Fe_2_^III^(TPA)_2_(μ-O)(μ-OAc)]^3+^ are identical within the statistical errors, as expected, since they were optimized on the basis of the same XANES spectrum.

## 4 Conclusions

This work demonstrates that it is possible to derive important mechanistic insights for a reactive process occurring in solution on the millisecond scale and to structurally characterize its transient intermediates through a multivariate EDXAS analysis. The implemented approach has enabled the direct determination of the mechanism of the reaction between [Fe^II^(TPA)(CH_3_CN)_2_]^2+^ and AcOOH using the TPA nonheme complex at 25 °C. In particular, it is confirmed that an Fe(III) acyl-peroxo intermediate is initially formed, which in turn evolves to a Fe(IV) oxo complex. The sixth ligand of the latter species, which was previously unidentified, is shown to be an acetate ion. This strategy allows one to characterize elusive intermediates whose geometries cannot be easily determined using the conventional experimental methods. Its combination with EDXAS holds great promise, especially for the investigation of complex redox reaction mechanisms on organic substrates that are silent to laboratory-based spectroscopies.
